# Uncertainty in Quantitative Electron Probe Microanalysis

**DOI:** 10.6028/jres.107.040

**Published:** 2002-12-01

**Authors:** Kurt F. J. Heinrich

**Affiliations:** National Institute of Standards and Technology, Gaithersburg, MD 10899-0001

**Keywords:** absorption coefficients, accuracy, microanalysis, models, x-ray absorption, x-ray spectrometry

## Abstract

Quantitative electron probe analysis is based on models based on the physics or x-ray generation, empirically adjusted to the analyses of specimens of known composition. Their accuracy can be estimated by applying them to a set of specimens of presumably well-known composition.

## 1. Correction Procedures

The doctoral thesis of Raymond Castaing [1] contains the outlines of a procedure of quantitative electron probe microanalysis (EPMA) analysis on which most subsequent methods are modeled. His choices were influenced by the availability of instrumentation and data. The instrument he used had crystal spectrometers of unnecessarily high wavelength resolution and inherent mechanical instability. His Geiger detectors had a very high dead time (in the order of ms) so that no high intensities could be accurately measured. The beam stability was not comparable to present standards, the vacuum was usually poor, and there were no diffracting crystals available for wavelengths below 0.1 nm. The efficiency of his instrument was relatively low so that he was forced to use acceleration voltages as high as 29 kV for routine analysis. There was at this time no energy-dispersive equipment available, and, last but not least, there existed no computers that would have permitted extensive on-line calculations or storage of parameters.

Since it was practically impossible to compare the generated intensities of x-ray emissions at different wavelengths, Castaing chose to compare the measured intensities of the same x-ray line from the specimen and a standard of known composition determined sequentially. [With modern energy spectrometers in which the efficiency change from one element to the next can be estimated accurately, quantitation by comparison of the intensity from several lines (“standardless analysis”) is now feasible]. Usually, pure elements were used as standards where possible.

Castaing recognized the existence of absorption effects in primary emission, of fluorescence due to characteristic lines, and of matrix effects (atomic number effects) in the primary emission. Hence he proposed three “corrections” to the measured intensity: for absorption, atomic number effect, and fluorescence (from characteristic lines only). It was impossible to predict quantitatively the intensity of primary emission or the signal losses due to the absorption of x-rays within the specimen. Only the relative contribution of fluorescence due to characteristic lines could be calculated from first principles. Fluorescence by the continuum was ignored, as were at first the effects of electron backscatter, and many parameters of importance in quantitative analysis, particularly the x-ray absorption coefficients that were not well known.

The most important effect to be accounted for was that of losses due to x-ray absorption, particularly significant because of the low take-off angle and the use of high acceleration voltages. In his thesis Castaing tried to obtain information concerning the depth distribution of primary x-ray generation, which must be known if the absorption losses are to be calculated. Later he continued this effort with the aid of special targets with thin tracer layers buried at varied depth [2,3]. Complementary information on this subject was obtained by Green [4] who measured the intensity of x-ray emission as a function of the x-ray emergence angle. Based on these observations, Philibert first proposed a generalized model for the calculation of the absorption losses of primary emission [5]. Further refinements of the “absorption correction” were later proposed by various authors [6]. The calculation required, however, a reasonably accurate knowledge of the x-ray absorption coefficients involved. This problem was tackled by experimental determinations as well as by the generalized models for the calculation of these coefficients [7].

The accuracy of these models and of the analyses performed with their aid is thus limited by the following factors:

1. X-ray intensity measurement uncertainties due to counting statistics, drift, dead-time corrections, and those relating to line and band widths.
2. Chemical shifts.
3. Uncertainties in physical parameters used in the correction procedure, such as mass absorption coefficients.
4. Limitations in the amount and type of composite standards used for the calibration of such procedures.
5. Uncertainties in chemical analysis, inhomogeneity of standards and specimens, and in the assumed stoichiometry of the standards.
6. Effects of standard preparation, surface conditions, poor conductivity, and specimen decomposition upon irradiation.

Mechanisms of x-ray generation of less importance, such as fluorescence due to the continuum, and excitation by high-energy secondary electrons [8] are usually ignored in the procedure. They may have been incorporated inadvertently in one of the classical corrections of the ZAF procedure. In that case, adding a separate calculation for them may actually degrade the accuracy of the procedure.

In view of the limited knowledge of the laws governing the generation of primary x-rays in multi-element targets, any “correction method” is, or should be, based on generalizing the results of analyses of specimens of known composition. The comparison of competing procedures is also done on the basis of applying them to measurements on sets of standards of “known” composition. Ideally one should evaluate a method with a set of standards that were not used for its creation, but this is virtually impossible, given the scarcity of measurements on reliable standards. The evaluation of the residual errors was usually done for the combined effects of atomic number, absorption and fluorescence, but obviously the tests for each of the corrections should be done separately for each effect. For instance, the inclusion in a test for absorption of specimens with atomic number differences but negligible absorption will increase the statistical uncertainty. Separate tests of this nature were described in a report on a study performed after my retirement, using an extended and carefully selected set of standard materials, and summarized in Ref. [9].

As an alternative procedure, particularly for minerals, composite standards of presumably known composition can be combined with simple correction procedures. In this case, the residual uncertainties are mainly due to the accuracy of the presumed standard composition, and on the macroscopic and microscopic homogeneity of the standards.

## 2. Contributions of NBS/NIST

Work done at NBS/NIST that contributed to the improvement of the accuracy of microanalysis included proposed models for data reduction programs, for the required parameters, and for their performance with computers. These contributions are too numerous to be detailed here.

Other publications were concerned with the way in which uncertainties in physical parameters affect the accuracy of the result [10–12]. The most significant effect of these studies was the demonstration that the accuracy of analysis could be improved significantly by using spectrometers at a higher take-off angle than Castaing’s original instruments, and working at lower operating voltages. These changes were adopted by all instrument manufacturers and analysts. Another area of importance was the preparation of standard reference materials certified for composition and homogeneity on a microscopic level and for particulate material [13].

Several workshops at NBS/NIST provided a basis for the collection of work of general interest, with the participation of investigators from abroad. The presentations are collected in NBS Special Publications [14–16] and in a book edited by Heinrich and Newbury [17].
